# Provincialising Global Oncology

**DOI:** 10.1136/bmjgh-2025-019950

**Published:** 2025-11-21

**Authors:** Nickolas Surawy Stepney, Shagufta Bhangu, Henry Llewellyn, Jennifer Fraser, Thandeka Cochrane, Philip Jagessar, David Reubi, Carlo Caduff, Freddie Bray, Nils Graber, Oliver Henke, Jorge Alberto Bernstein Iriart, Benson A Azariah Mulemi, Robert Newton, Ruth Prince, Clémence Schantz, Manju Sengar, Bhawna Sirohi

**Affiliations:** 1 University of Exeter, Exeter, UK; 2 King’s College London, London, UK; 3 King's College London, London, UK; 4 International Agency for Research on Cancer, Lyon, Auvergne-Rhône-Alpes, France; 5 Humbolt University, Berlin, Germany; 6 Kilimanjaro Christian Medical Centre, Moshi Urban, Tanzania, United Republic of; 7 Instituto de Saúde Coletiva, Federal University of Bahia, Salvador, Bahia, Brazil; 8 University of Pretoria, Pretoria, South Africa; 9 Uganda Virus Institute, Entebbe, Uganda; 10 University of Oslo, Oslo, Oslo, Norway; 11 Ceped UMR 196, Institut de recherche pour le developpement, Paris, France; 12 Tata Memorial Hospital, Mumbai, MH, India; 13 BALCO Medical Centre, Raipur, India

**Keywords:** Cancer, Global Health

**Summary**

- Global Oncology is an important project and concept which has made significant strides in recent years. Its laudable achievements in tackling cancer’s medical and human impacts have worked to address the ‘oncological divide’.
- To strengthen the project’s core aims, we call to ‘provincialise’ its goals and techniques by bringing in voices of experts on the ground, in particular from the Global South.
- This is crucial to strengthening Global Oncology, setting its agendas, suturing its standards and norms with on-the-ground realities, and considering complex and diverse dynamics of difference and inequality.
- Through provincialising, we advocate for a move towards plural oncologies.

‘Global Oncology’ has become an increasingly prevalent term in recent years. Arising from a renewed application of the language and theories of globalisation, which were so influential to HIV/AIDS and other infectious diseases in the 1990s and early 2000s, it is a term that means different things to different people.^1^ However, to its main exponents, it is generally understood as a set of interventions that aim to ‘close the divide’ in ‘cancer access and outcomes’ by addressing ‘disparities and differences in cancer prevention, care, research, education and the disease’s social and human impact around the world’.^2 3^

Global Oncology has helped draw attention to rising cancer rates and the difficulties of accessing oncological care in low-income and middle-income countries. It has also generated goodwill and raised funds to train oncologists in the Global South and equip them with expensive drugs, pathology laboratories and radiotherapy machines to care for their patients.^4^ There is no doubt that Global Oncology’s efforts to bring oncology to ‘millions of underserved human beings’ across the Global South are morally laudable and medically important.^5^

Nevertheless, a number of more controversial assumptions and limitations of Global Oncology remain relating to how its proponents envision and locate the ‘oncological divide’ and how they plan to close it. Drawing on a recent workshop (‘The Social and Political Lives of New Cancer Technologies in the Global South’, Brocher Foundation, Switzerland, June 2024) that brought together historians and social scientists with epidemiologists and oncologists from across the world, we highlight three principal challenges to the field: the overwhelming dominance of Global North countries in setting the terms of Global Oncology, its aspirations and curricula; the imposition of standards, tools and procedures that are often out of touch with situations on the ground; and a characterisation of global cancer care which obscures more complex dynamics of difference and inequality.

Global Oncology emerged as a field of practice, training and research in the 1990s and 2000s in academic and healthcare settings of the Global North marked by initiatives such as the 2012 Harvard Global Oncology Summit.^2^ Today, it remains concentrated in teaching centres, research institutes and publication platforms across North America and Europe, supported by well-resourced and powerful universities, charities and organisations like the American Society of Clinical Oncology. As a result, physicians from LMICs often have a marginal involvement in research planning and design, participating merely in the implementation of the projects.^1^ This has also entrenched North American and European standards and ideas about cancer care in global oncology curricula which include fundamental moral principles about *how to care* and *when to treat*.^6^

Relatedly, Global Oncology programmes have often imposed tools and procedures on Global South countries that are not always commensurate with existing health infrastructures or financial capabilities. Requirements for certain diagnostic tests to be performed before treatment, for example, have challenged or excluded places with limited or absent laboratory facilities. High technologies like radiotherapy machines are provided, which then fall out of service contracts, quickly becoming obsolete and unusable.^7 8^ Surgical and pharmaceutical interventions are supported and offered free to patients, frequently without similarly funded patient transportation, accommodation or ancillary care, making it available in practice only to those with means.^9^

More fundamentally, Global Oncology has tended to characterise global cancer care in a starkly bifurcated way. High-income countries are framed as places of affluence with world-leading oncological care for everyone, while low-income and middle-income countries appear as places of lack, where oncology is under-developed and unavailable for most. This not only contains assumptions about who should learn from whom and who needs to catch up with whom, but it also obscures much more complex and heterogenous geographies of inequality, care and collaboration.^10^ Research has shown a significant lack of access and equity of care in many high-income countries. Resources and expertise tend to be concentrated in specific areas within countries, and vast differences in care are experienced by those able to afford the most advanced treatments and those with little access to local medical care. Canada and the USA are key examples of this internal variation; but so are India and Brazil. Similarly, there are momentous inequalities within and between Global South countries. For example, oncology infrastructures vary greatly between India and Brazil, with their cutting-edge hospitals, vibrant oncological communities and innovative pharmaceutical industries, and Haiti and Mali, which have highly precarious and seriously under-resourced healthcare facilities. A new vision of the world is needed to account for complex realities that simple North/South distinctions fail to capture.

The effects of these challenges are myriad and manifest in relations among patients and providers, professional opportunities for clinicians and a whole range of other things, as seen in global responses to HIV/AIDS and other infectious diseases in an earlier era.^11^ At stake is the exacerbation of inequalities within and between countries, the displacement of expertise and practices that have developed within local contexts and dashed hopes when promises fail to materialise. One form this displacement takes is a concentration of Global South healthcare workers and expertise in Global North countries, resulting in a detrimental ‘brain-drain’. However, even when experts return to countries in the South, these actors do not necessarily usher in a change of oncology agendas but may be more inclined to perpetuate existing frameworks and expertise set in the North.

More recent Global Oncology initiatives raise new hopes, as we heard during our workshop. Professionals from the Global North are becoming more cognisant of the issues we highlight, seeking to mitigate them through more balanced collaborations and greater attunement to the social and material contexts in which they work.^5 12^ Global South actors are increasingly recognising their critical role in shaping or rejecting collaborations that do not meet their needs. This influence, however, is typically limited to established experts and institutions well-connected within international research and healthcare networks. Furthermore, even as specific collaborations are negotiated, the underlying aspirations and forms of cancer care in the Global South often perpetuate the values and terms of a globalised oncology model. This is partly due to the universalising nature of modern biomedical science, which is often deeply embedded in national discourses and state-led projects that dictate funding and investment priorities. There is also a growing recognition of partnerships among Global South countries themselves. These collaborations, often overlooked, are proving very effective at building appropriate oncology infrastructure and widening access to care.^13 14^

We argue that these initiatives, and the ethics that underwrite them, can no longer remain marginal to the overall framing, purpose and orientation of Global Oncology; they must instead be placed front and centre. This means reimagining and decentring the Global North as the leader and sole provider of good oncological care. It also means scrutinising assumptions about the nature and location of ‘divides’ that have reified the Global North as the place of plenty and the Global South as the impoverished ‘other’. And it means ridding the field of the assumption that the divide can be bridged only by extending those same technologies and standards from the North to the South.

In short, what we need is not the spread of a singular, monolithic ‘global oncology’, but numerous different ‘oncologies’, embedded in and cognisant of the epistemologies, politics and care infrastructures of individual locations. Whereas recent critics of Global Oncology have argued that the future of the field is one ‘beyond borders’^1^ —a universalising phenomenon that unites diverse communities in the common vision of reducing equity gaps—we encourage policymakers and physicians to ‘provincialise oncology’^15^ —to locate it within a particular place and context, and rob it of its supposed universal or transcendental character as the *only* oncology. By doing this, we can better understand and strengthen the politics, epistemics and practices of the field.

## Data Availability

There are no data in this work.
